# The Tumor and Host Immune Signature, and the Gut Microbiota as Predictive Biomarkers for Immune Checkpoint Inhibitor Response in Melanoma Patients

**DOI:** 10.3390/life10100219

**Published:** 2020-09-25

**Authors:** Katarzyna Tomela, Bernadeta Pietrzak, Marcin Schmidt, Andrzej Mackiewicz

**Affiliations:** 1 Department of Cancer Immunology, Chair of Medical Biotechnology, Poznan University of Medical Sciences, 8 Rokietnicka Street, 60-806 Poznan, Poland; mackiewicz.aa@gmail.com; 2 Department of Food Biotechnology and Microbiology, Poznan University of Life Sciences, 48 Wojska Polskiego Street, 60-627 Poznan, Poland; bernadeta.pietrzak@up.poznan.pl (B.P.); marcin.schmidt@up.poznan.pl (M.S.); 3 Department of Diagnostics and Cancer Immunology, Greater Poland Cancer Centre, 15 Garbary Street, 61-866 Poznan, Poland

**Keywords:** biomarkers, immunotherapy, immune checkpoint inhibitors, gut microbiota, melanoma

## Abstract

There are various melanoma treatment strategies that are based on immunological responses, among which immune checkpoint inhibitors (ICI) are relatively novel form. Nowadays, anti-cytotoxic T-lymphocyte-associated protein 4 (CTLA-4) and anti-programmed death-1 (PD-1) antibodies represent a standard treatment for metastatic melanoma. Although there are remarkable curative effects in responders to ICI therapy, up to 70% of melanoma patients show resistance to this treatment. This low response rate is caused by innate as well as acquired resistance, and some aspects of treatment resistance are still unknown. Growing evidence shows that gut microbiota and bacterial metabolites, such as short-chain fatty acids (SCFAs), affect the efficacy of immunotherapy. Various bacterial species have been indicated as potential biomarkers of anti-PD-1 or anti-CTLA-4 therapy efficacy in melanoma, next to biomarkers related to molecular and genetic tumor characteristics or the host immunological response, which are detected in patients’ blood. Here, we review the current status of biomarkers of response to ICI melanoma therapies, their pre-treatment predictive values, and their utility as on-treatment monitoring tools in order to select a relevant personalized therapy on the basis of probability of the best clinical outcome.

## 1. Introduction

Melanoma is a skin cancer that arises from the transformed melanocytes. Among skin cancers, malignant melanoma has the highest morbidity rate [1]. According to The Global Cancer Statistics there were 287,723 new melanoma cases and 60,712 deaths worldwide in 2018 [2]. Early diagnostic and an effective melanoma treatment strategy are crucial for increasing survival rates of melanoma patients. There are various melanoma therapies, such as surgical resection, chemotherapy, biochemotherapy, photodynamic therapy, immunotherapy, or targeted therapy that are introduced as monotherapies or combined therapies. Selection of a relevant therapeutic strategy depends on the patient’s clinical condition, cancer stage, and location [1].

Mutations in the B-Raf proto-oncogene (BRAF) generate activation of the mitogen-activated protein kinase (MAPK) signaling pathway and increase growth and proliferation of cancer cells [3]. Patients diagnosed with tumor BRAF mutation undergo targeted therapy with a BRAF inhibitors (vemurafenib or dabrafenib) in the first line of treatment [4]. Currently, immunotherapy represents a standard treatment approach in metastatic and unresectable melanoma. Immunotherapy mechanism relies on the interaction between immune system and surface molecules on cancer cells [5]. There are various melanoma treatment strategies that are based on immunological responses, among which immune checkpoint inhibitor (ICI) therapy is a novel form.

Tumor cells activate immune checkpoint pathways to evade elimination mediated by immune cells. The mechanism of ICI therapy against cancer is based on blocking their suppressive effects and promoting antitumor immune responses [6]. In 2018, James P. Allison and Tasuku Honjo received the Nobel Prize in Physiology or Medicine for the discovery of immune checkpoint pathways [7]. Allison et al. conducted studies on cytotoxic T-lymphocyte-associated protein 4 (CTLA-4) and developed a cancer treatment strategy based on CTLA-4 blockade with anti-CTLA-4 antibodies [8]. The CTLA-4 is expressed on regulatory T-cells (Tregs) and exhausted T-cells. It represents a high affinity binding receptor for CD80 and CD86 that are present on the surface of antigen-presenting cells (APCs). The CTLA-4 binding to its ligands inhibits activation of the signaling pathway and increases T-cell activation threshold. Consequently, responses to self and tumor antigens are suppressed [9]. Ipilimumab, the commercially available anti-CTLA-4 antibody, blocks CTLA-4 and restores activation of effector T-cells and their proliferation [6]. Clinical trials demonstrating the antitumor activity of ipilimumab resulted in its approval by the U.S. Food and Drug Administration (FDA) for the treatment of advanced melanoma in 2011 [1,5]. Moreover, Honjo and his collaborators discovered another immune checkpoint receptor, known as programmed death-1 (PD-1) receptor, which is expressed on T-cells, B-cells, dendritic cells (DCs), natural killer (NK) cells, and Tregs [7,10,11]. Different types of cancer cells express PD-1 ligands, such as programmed death-ligand 1 (PD-L1) and PD-L2, which bind to PD-1 and inhibit signaling pathways, leading to T-cell exhaustion and suppression of immune-mediated antitumor responses [11]. Understanding of the PD-1 immunosuppressive mode of action resulted in the selection of a new target for cancer ICI therapy. Nivolumab and pembrolizumab are anti-PD-1 antibodies that were approved by the FDA for the treatment of advanced melanoma in 2014 and 2015, respectively. They bind to the PD-1 receptor on the T-cell surface and block its interaction with PD-L1 and PD-L2, thus preventing immune suppression stimulated by cancer cells [1].

Although there are remarkable curative effects in patients who respond to ICI therapy, up to 70% of melanoma patients show resistance to this treatment [12]. One study demonstrated that long-term clinical outcome was observed in 22% of melanoma patients treated with ipilimumab [13]. Similarly, another study indicated that barely 33% of melanoma patients treated with pembrolizumab showed response or cancer control, whereas durable effects were observed in about 80% of them [13,14]. Low response rate to ICI treatment is caused by innate as well as acquired resistance. Some aspects of treatment resistance are still unknown, however, cancer progression development in initially responding patients might be associated with numerous factors, such as loss of neoantigen expression, inhibition of immune signaling pathways, or impaired formation of memory T-cells [13].

Consequently, novel treatment strategies are explored to overcome treatment resistance. These are, for instance, identification of alternative inhibitory receptors, examination of other antibodies targeting immune checkpoints, and combined therapies administration [6,9]. In order to find novel inhibitory receptors, there are studies conducted on receptors expressed on immune cells as well as cancer cells [5,9]. Given as an example, atezolizumab that targets PD-L1 on cancer cells is studied as a potential antibody for melanoma ICI therapy [11]. Simultaneously, numerous clinical trials are carried out to estimate the efficiency of other antibodies targeting immune checkpoints [9]. Moreover, advanced melanoma treatment includes a combination of therapies to enhance their efficacy. There are several treatment strategies, for instance, ICI therapy is administered along with other antibodies targeting PD-1 or CTLA-4, with other immunotherapy, chemotherapy, or targeted therapy [5,15].

The increasing number of therapeutic approaches for melanoma patients leads to a need for establishing predictive biomarkers to select a relevant personalized therapy, on the basis of probability of the best clinical outcome. Every therapeutic intervention, including ICI therapies, is related to a risk of developing some adverse effects. In some cases, ICI therapies may increase activity of the immune system and induce inflammation, which can be responsible for developing immune-related adverse events (irAEs). The most frequently reported irAEs in melanoma patients are skin rash and pruritus (30–55%); gastrointestinal events such as diarrhea and colitis (12–37%); and hepatic, endocrine, and neurological symptoms. Combined therapy with nivolumab and ipilimumab generates a higher risk of developing severe toxicities compared with monotherapies (ipilimumab, nivolumab, or pembrolizumab) [16,17]. Therefore, establishing predictive biomarkers could abolish side effects related to ineffective therapy, and instead offer a different option as the first-line treatment. Moreover, immunotherapy generates very high costs, and therefore accurate drug administration would have a positive impact on the health economy.

**Figure 1:**
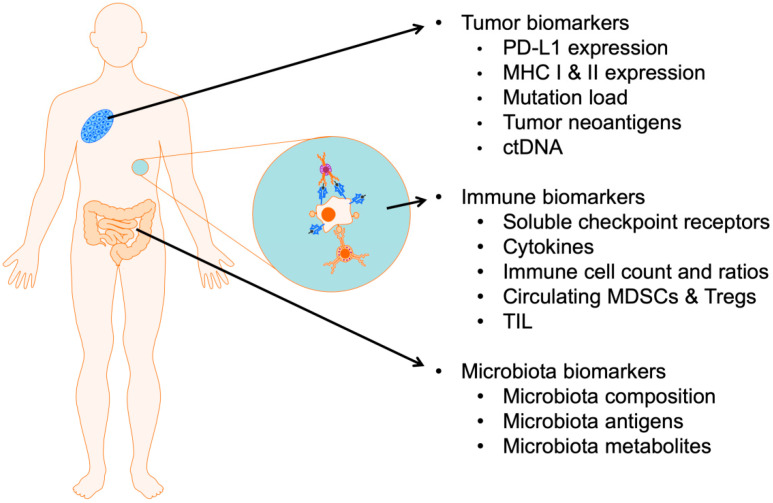
Summary of reviewed biomarkers of response to immune checkpoint inhibitor (ICI) therapies for melanoma patients. PD-L1: programmed death ligand 1, MHC I: major histocompatibility complex class I, MHC II: major histocompatibility complex class II, ctDNA: circulating tumor DNA, MDSC: myeloid-derived suppressor cells, Tregs: regulatory T-cells, TIL: tumor infiltrating lymphocytes.

Biomarkers are classified into prognostic and predictive forms. Prognostic biomarkers are related to parameters, values, or tendencies that describe the general stage of disease and clinical characteristics, as well as being able to provide information about the overall disease outcome. In contrast, predictive biomarkers provide insight into the probability of benefitting from particular treatment. Pre-treatment predictive biomarkers would be helpful in deciding on the type of therapy. Biomarkers analyzed prior to treatment would provide early indicators of response or progression. In this review, we discuss several biomarkers for predicting the response to ICI treatment in melanoma patients from three different perspectives: (i) that related to molecular and genetic tumor characteristics, (ii) that related to the host immunological response, and (iii) that related to the host gut microbiota. The described biomarkers are summarized in the table (Table 1). Furthermore, we describe herein pre-treatment predictive values of biomarkers and their utility as on-treatment monitoring tools (Figure 1).

## 2. Predictive Biomarkers of Response Related to the Tumor

Some of the basic clinical tumor parameters such as tumor burden or metastatic site are not only prognostic but have also been found to be predictive biomarkers for the clinical outcome of ICI therapies. For example, melanoma patients with lung and skin metastases were reported to achieve favorable overall survival (OS) in response to anti-PD-1 therapy compared to patients with metastases in other organs [18]. Moreover, baseline tumor size (BTS) below the median is associated with higher overall response (ORR) and improved OS in advanced melanoma patients treated with pembrolizumab [19]. Tumor biopsies provide the most direct information about cancer characteristics and are a wide source of already established and potential biomarkers of response to ICI therapies. However, it was reported that expression level of immune checkpoint molecules can differ between tumor lesions, primary tumors, and metastases due to tumor heterogeneity [20]. Moreover, availability of such samples is limited during treatment, and thus their application as an on-treatment monitoring of response to therapy is generally not possible. This section provides a review of the recent literature on several potential biomarkers of response to ICI therapies related to the tumor characteristics and oncogenic adaptive immune resistance mechanisms.

### 2.1. PD-L1 Expression on Tumor Cells

Intracellular expression of PD-L1 (B7-H1, programmed death-ligand 1) in tumors has been reported in various carcinomas such as melanoma, non-small-cell lung carcinoma (NSCLC), renal cell carcinoma (RCC), and ovarian and colorectal cancers. Some tumors such as melanoma and NSCLC have been shown to express PD-L1 more abundantly than others. Interestingly, PD-L2 and PD-1 are also present in tumors, especially on cells related to tumor microenvironment rather than on tumor cells themselves [21,22]. PD-L1 expression is observed on macrophages and DCs to a limited extent, as well as on many other cell types with inflammation, and can also be induced by different factors. Blockade of PD-1/PD-L1 synapse results in inhibition of activated T-cells and further T-cell exhaustion, which has a positive impact on downregulation of responses in autoimmune diseases but also weakens anti-cancer response [23]. Tumor cells have developed adaptive immune resistance to avoid antitumor immune responses, which relies on PD-L1 expression stimulated by inflammatory signals such as interferon gamma (IFN-γ) secreted by activated tumor-infiltrating cells [24,25].

Taube et al. [21] reported a positive correlation between PD-L1 expression on pre-treatment tumor cells biopsied from melanoma, NSCLC, and RCC patients and objective response, as well as the occurrence of clinical benefit from nivolumab treatment. Moreover, PD-L1 was detected on tumor-infiltrating cells and was correlated with a clinical benefit, but not with clinical response to anti-PD-1 therapy. Similar results were obtained previously by Topalian et al. [26], who showed that patients with various cancers with PD-L1 expression were more likely to obtain an objective response (36%, *p* = 0.006) and that PD-L1 expression below cut-off (<5%) was related to no response to anti-PD-1 therapy. Daud et al. [27] performed PD-L1 expression analysis in pre-treatment tumor biopsies from 405 melanoma patients enrolled in the KEYNOTE-001 trial treated with pembrolizumab. PD-L1 expression was correlated with a better response rate, progression-free survival (PFS), and OS. However, some patients with PD-L1-negative tumors also benefitted from therapy, achieving durable responses.

The utility of PD-L1 tumor status in predicting response to nivolumab and nivolumab plus ipilimumab combined therapy was analyzed in tumor pre-treatment biopsies from melanoma patients enrolled in the CheckMate 067 trial. A better survival outcome was observed in a group of patients subjected to combined therapy and whose tumors were expressing PD-L1 at a low level. However, OS did not differ significantly between patients receiving monotherapy or combined therapy with a ≥1% or ≥5% tumor PD-L1 expression. The ORR was higher in the combined nivolumab/ipilimumab patients group and was not associated with a PD-L1 expression level on tumor cells, revealing a poor predictive potential of PD-L1 tumor expression for anti-PD-1 and anti-CTLA-4 combined therapy [28].

Several factors affect the usefulness of PD-L1 immunohistochemistry (IHC) as a biomarker for ICI therapies. Firstly, various assays are in use and divergent thresholds of PD-L1 positivity and cut-off values are set. Secondly, PD-L1 is heterogeneously expressed within a tumor mass, and thus it may be inapplicable in primary melanoma and distant metastases evaluation [20,29]. Moreover, PD-L1 expression is regulated by various mechanisms, and its level can change in the time between the sampling of biopsy and the therapy administration [30]. Indeed, some anti-cancer therapies were reported to affect PD-L1 expression [31]. Finally, patients with negative PD-L1 tumors may also respond to anti-PD-1 therapy [27]. Nevertheless, the PD-L1 IHC test was recommended by the FDA as a tool for stratifying NSCLC patients before pembrolizumab therapy because of the strong correlation of tumor PD-L1 expression (over 50% cells expressing PD-L1) and higher response rate, as well as prolonged PFS and OS [32].

### 2.2. MHC I and II

The expression of major histocompatibility complex class I and II (MHC I and MHC II) is essential for antigen presentation. MHC I presents antigen to CD8⁺ cytotoxic T-cells (CTLs) and is abundantly expressed in different types of cells, whereas MHC II presents antigen to CD4⁺ T-helper cells and is restrictedly expressed on professional APCs such as DCs or B-cells [33,34]. Previous studies demonstrated a relationship between MHC I and II downregulation and tumor progression, with a worse prognosis in many malignancies, including melanoma. MHC I/II downregulation was hypothesized to be one of the mechanisms of avoiding immune recognition by tumor cells [35,36,37].

Johnson et al. [38] analyzed the level of MHC class II cell surface receptor- human leukocyte antigen—DR isotype (HLA-DR) expression in tumor biopsies from melanoma patients treated with anti-PD-1 or anti-PD-L1 therapy and reported the association of a high level of MHC II (HLA-DR) expression with improved clinical response, as well as longer PFS and OS, compared to patients with a low MHC II expression. The acquired resistance to anti-PD-1 immunotherapy in melanoma tumors was found to be related to several mutations in genes encoding interferon receptor-associated Janus kinase 1 (JAK1) and Janus kinase 2 (JAK2), which could result in the insensitivity of tumor cells to IFN-γ. Another mutation identified in tumor biopsies of non-responders was the mutation in antigen-presenting protein beta-2-microglobulin (B2M), one of the components of MHC I. This aberration can lead to a loss of surface expression of MHC I and disable a proper CD8⁺ T-cell recognition [39].

In a more recent study, Rodig et al. [40] examined MHC I and II protein expression in the pre-treatment biopsies from melanoma patients enrolled in the CheckMate 064 (ipilimumab vs. nivolumab) and CheckMate 069 (ipilimumab monotherapy vs. combined therapy with ipilimumab plus nivolumab) trials and compared them with a clinical outcome observed within 13 weeks of treatment. Reduced MHC I expression in tumors (≤30%) was reported to be a negative predictive marker of response to ipilimumab and was associated with the worst OS and lack of clinical response, but was not related to clinical benefit of nivolumab therapy. On the other hand, tumoral MHC II expression (>1%) was associated with the occurrence of complete or partial response and stabilization of disease in a group receiving nivolumab, but not ipilimumab. Interestingly, the clinical outcome from combined therapy was not related to MHC I or MHC II tumor expression. Altogether, these results show a significant correlation between primary resistance to anti-CTLA-4 or anti-PD-1 and tumor MHC I/II expression, which has a strong potential as a predictive biomarker in ICI therapies.

### 2.3. Mutational and Neoantigen Load

Among various cancer types, melanoma and NSCLC have the highest median mutational load. Moreover, the patient’s response to ICI therapies is more frequent compared to patients with low mutational load cancers, such as pancreatic and prostate cancer [26,41]. The increased mutation load could be a result of mutations in specific oncogenes and tumor suppressor genes, or somatic mutations. The median mutational load in smoking-associated lung cancer is higher than in non-smoking-associated lung cancer, and smoking patients have been reported to have a better response rate to pembrolizumab therapy [32,42]. An intriguing observation was made in melanoma patients under combination therapy with ipilimumab followed by radiation treatment. Postow et al. showed that irradiation of one lesion resulted in the regression of metastatic lesions at a distance, suggesting that mutation-inducing therapy enhanced the effectiveness of anti-CTLA-4 agents [43] as a result of a so-called abscopal effect.

An elevated mutation load was postulated to increase the production of new proteins expressed on the tumor cell surface as neoantigens. Different neoantigens could generate an additional pool of tumor-specific T-cells, thus increasing tumor recognition and efficacy of ICI [44]. In the support of this hypothesis, melanoma responders to anti-CTLA-4 therapy were shown to produce T-cells that are specific for neoantigens related to tumor somatic mutations [45].

A whole-exome sequencing (WES) of baseline tumor biopsies from 64 melanoma patients receiving CTLA-4 inhibitors (ipilimumab or tremelimumab) revealed over 100 somatic mutations related to a degree of clinical benefit within 6 months of therapy in responding patients (*p* = 0.01). Moreover, a pool of the neoantigens related to MHC I associated with the response to anti-CTLA-4 agents and survival was identified [46]. Further analysis performed by van Allen et al. [47] supported the predictive value of clinical benefit related to mutational load in melanoma tumors of responders to CTLA-4 inhibition. However, in the case of neoantigens, van Allen et al. noticed a high variety of antigens among melanoma tumors and revealed that neoantigen specificity was more related to the host than the tumor itself. As a result, this finding had no predictive value for response to ICI therapy, even though a load of neoantigens was elevated in responding patients.

Johnson et al. [48] analyzed 65 archival samples from melanoma patients by hybrid-capture-based next-generation sequencing (NGS). Responders to anti-PD-1 were reported to have a higher mutational load; moreover, this elevated mutation load was associated with a longer median PFS and OS compared to non-responders. Contrarily, Hugo et al. reported that although a high mutation load was associated with prolonged OS of patients receiving anti-PD-1 (pembrolizumab or nivolumab), it was not related to the tumor response. WES analysis of 38 pre-treatment melanoma biopsies identified increased neoepitope loads in responding tumors, but changes did not reach statistical significance [49].

Altogether, these results demonstrate that mutation and neoantigen load could have a prognostic and predictive value for clinical response to ICI therapies. However, there are some limitations for practical application in clinical diagnostics. They are mainly related to high costs of whole-exome sequencing and difficulties in the interpretation of data, especially since the part of neoantigens might be patient-related. Therefore, prior development of a neoantigen database is required.

### 2.4. Liquid Biopsy

Liquid biopsy is a non-invasive method of detection and monitoring of tumor-derived compartments in patients’ blood, such as circulating tumor DNA (ctDNA) and circulating tumor cells (CTC). Together with a conventional tumor biopsy, liquid biopsy provides comprehensive information about tumor characteristics and disease development. Moreover, in contrast to tumor mass biopsy, it enables a regular sampling to define patient response status. For instance, a high pre-treatment ctDNA level in blood was reported as a biomarker of tumor burden and was associated with a poorer prognosis for cancer [50]. Furthermore, ctDNA in peripheral blood is directly related to the tumor, unlike many other molecular biomarkers.

Cabel et al. [51] compared ctDNA levels in 15 NSCLC, uveal melanoma, and microsatellite-unstable colorectal cancer patients before receiving nivolumab or pembrolizumab and during therapy. In the serum of 10 patients, the authors did not detect ctDNA at the baseline, which was associated with improved OS (hazard ratio (HR) = 6.8, 95% CI 1.1–41, *p* = 0.03), but not with PFS. Decrease of ctDNA to undetected level at week 8 compared to detectable level was associated with significantly better PFS (median 11 vs. 2 months, *p* = 0.004) and OS (HR = 15, 95% CI 2.5–95, *p* = 0.004). These results are in agreement with findings of Gray et al. [52], who analyzed ctDNA levels in 19 melanoma patients treated with ICI therapies (ipilimumab, nivolumab, pembrolizumab, or ipilimumab combined with pembrolizumab). Lower baseline ctDNA and a decrease of ctDNA during 4–8 weeks immunotherapy were associated with a better response and prolonged PFS (over 6 months) in all therapies. However, these results are unlikely to provide a predictive biomarker of response due to the use of small patient groups, the different types of cancer investigated, and the variable treatment options. Conversely, Serement et al. [53] performed a study on 85 melanoma patients receiving anti-PD-1 immunotherapy in which plasma cfDNA was analyzed for *BRAF*^V600^ and proto-oncogene *NRAS*^Q61/G12/G13^ mutations. Patients with undetected ctDNA at the baseline were reported to have a better clinical outcome: better PFS (median 26 weeks vs. 9 weeks, *p* = 0.01) and better OS (median not reached vs. 21.3 weeks, *p* = 0.005) compared to patients with a detectable ctDNA. Moreover, >500 ctDNA copies detected before treatment or as early as the third week of therapy were related to a poor clinical outcome following anti-PD-1 therapy. Interestingly, the ctDNA level was not elevated in the serum of patients with a progression exclusively in the central nervous system [52,53]. These results show limitations of ctDNA prognosis application for brain metastases, mainly as a result of blood–brain barrier selectivity [54].

Analysis of ctDNA blood serum level can be also applied to discern between pseudoprogression and true progression. For instance, in the study of 125 melanoma patients receiving anti-PD-1 monotherapy or anti-PD-1 combined with ipilimumab, at week 12 of therapy, the 29 patients were defined as progressive (according to Response Evaluation Criteria in Solid Tumors, RECIST). In the 29 patients who progressed during treatment, 20 had a true progression and nine had a pseudoprogression. All patients with a pseudoprogression represented a ctDNA profile corresponding to tumor regression, such as undetectable ctDNA at baseline or decrease in ctDNA after 12 weeks of therapy. In contrast, 90% of patients with a true progression had unfavorable ctDNA profiles (detectable ctDNA at baseline and stable or increased during treatment) [55]. These results prove that ctDNA analysis could be a relevant tool for the diagnosis of pseudoprogression that was reported to occur in up to 10% of patients treated with anti-PD-1 therapy [56].

## 3. Predictive Biomarkers Related to the Host Immune System

Immune system biomarkers provide the most desired type of markers because of easy specimen collection and availability of assays. The biomarkers or diagnostic methodology are mostly readily available in most clinical laboratories and do not require bioinformatic data curation or interpretation. This section lists several potential biomarkers of response to ICI therapies that are related to the host immune system and are detected in patients’ blood. However, immune cells are also present in tumor tissues. High tumor-infiltrating lymphocytes (TIL) grade in melanoma biopsies have been associated with a lower risk of death for patients with primary and metastatic melanoma, regardless of the type of treatment [57]. Recruitment of cytotoxic T-cells to the tumor microenvironment is an important factor of antitumor activity of the host immune system in response to ICI agent therapy. Tumeh et al. [58], in a retrospective study, analyzed tumor biopsies from 46 melanoma patients before and during therapy with pembrolizumab. Higher TIL density in pre-treatment samples was observed in responders compared to non-responders; moreover, during treatment proliferation of CTLS in invasive tumor margin was correlated with a reduction of tumor size upon pembrolizumab exposure in the responding group of patients. More recently, Vilain et al. [59] reported a sevenfold higher intratumoral PD-1⁺T-cell density in pre-treatment tumor biopsies from melanoma responders to anti-PD-1 therapy (pembrolizumab or nivolumab) compared to non-responders (*p* = 0.006). During treatment, a significant increase of TIL was observed in responders’ biopsies compared to pretreatment samples (*p* = 0.046), highlighting an importance of intratumoral T-cell infiltration in tumor shrinkage and response to anti-PD-1 therapy.

### 3.1. Immune Regulatory Molecules

#### 3.1.1. LDH, CRP, and S100B

Serum lactate dehydrogenase (LDH) and C-reactive protein (CRP) are standard blood parameters that are routinely tested during treatment. Together with cytoplasmic S100 calcium-binding protein B (S100B), they are well-known prognostic biomarkers of response to ICI therapy. Elevated LDH level is one of the markers of tumor burden and a negative prognostic factor for metastatic melanoma patients [19]. The American Joint Committee on Cancer (AJCC) included LDH as one of the elements of staging guidelines for melanoma patients with distant metastases [60].

Several studies showed the association between an elevated basal level of LDH and worse outcome of ICI therapy for melanoma patients, including ipilimumab, anti-PD-1, or combined (nivolumab together with ipilimumab) therapies. Moreover, the increase of LDH level during therapy was reported as an early negative predictive biomarker related to shorter OS [61,62,63,64,65]. Interestingly, an elevated LDH level was also observed in some patients who had responded to treatment, showing that LDH level should not be used as an excluding factor for ICI therapies [61], [64,66].

CRP is directly related to the inflammatory status of patients. A higher CRP level is the independent negative prognostic biomarker for melanoma patients related to poor OS, compared to patients with normal CRP levels [67]. Simeone et al. [68] reported the association of a decreased CRP level with disease stabilization and survival during 12 weeks of ipilimumab treatment in melanoma patients. These results have confirmation in the work of Nyakas et al. [69], showing that increased CRP levels at several time points (baseline, week 4, and week 7) were observed in non-responders for ipilimumab therapy. Elevated pre-treatment CRP level was also shown to be related to poor OS in both anti-PD-1 and anti-CTLA-4 melanoma therapies [69,70].

S100B protein is another circulating serum factor with prognostic value for melanoma patients as its concentration reflects the response to treatment or tumor growth related to progression [71]. Serum S100B level was reported as a prognostic marker for response to pembrolizumab and combined nivolumab and ipilimumab therapy in melanoma patients. Importantly, elevated basal S100B level was associated with impaired OS, regardless of treatment. The increase of S100B level during the first 6 weeks of treatment was also associated with OS of patients subjected to anti-PD-1 and combined therapies [63].

#### 3.1.2. Cytokines

Cytokines are crucial regulatory molecules released in response to cellular stress related to infections but also to inflammation caused by carcinogens [72]. Patient cells compete with cancer cells for host-derived cytokines that affect tumor but also promote its growth. Consequently, the cytokine concentration in the baseline and during treatment is a potential source of biomarkers of response to ICI therapy [73].

Yamazaki et al. [74] reported that higher serum pre-treatment levels of IFN-γ, interleukin 6 (IL-6), and IL-10 were associated with tumor response to nivolumab in melanoma patients and that decreased levels of these cytokines were observed in non-responders. IFN-γ and IL-6 are proinflammatory cytokines and their elevated concentration in ICI responders could reflect spontaneous anti-tumor immune response, however, the explanation for higher IL-10 levels is much more obscure. As an immunosuppressive cytokine related to the tumor microenvironment, the elevation of IL-10 in ICI therapy might be caused by tumor evasion mechanisms that suppress the activation of T-cells.

There is strong evidence for the positive correlation between elevated levels of pro-inflammatory cytokines and the occurrence of adverse events during ICI treatment. Tanaka et al. [75] recorded the correlation between an elevated baseline IL-6 level and irAE in a small group of melanoma patients treated with nivolumab. Moreover, elevated expression of 11 cytokines: granulocyte colony-stimulating factor (G-CSF), granulocyte-macrophage colony-stimulating factor (GM-CSF), Fractalkine, fibroblast growth factor 2 (FGF-2), IFN-α2, IL-12p70, IL-1a, IL-1B, IL-1RA, IL-2, and IL-13) were associated with severe immune-related toxicity in melanoma patients treated with combined anti-CTLA-4 and anti-PD-1 therapies [76].

#### 3.1.3. Soluble Checkpoint Molecules

Soluble programmed death ligand 1 (sPD-L1) is a circulating form of PD-L1 that has a predominant membrane-bound PD-L1 form (mPD-L1). The precise origin and biological activity of sPD-L1 are still not completely understood. Chen et al. [77] showed that sPD-L1 is released by PD-L1-positive cells to culture medium by a proteolytic disruption catalyzed by a metalloproteinase activity. Alternative splicing of transcript encoding PD-L1 was also reported as a source of sPD-L1 protein form [78,79,80]. Splice variants of PD-L1 lose the transmembrane or intracellular domain following alternative splicing and are secreted as sPD-L1 [81]. Interestingly, patients with solid tumors were reported to have a higher sPD-L1 level than healthy controls [81,82]. Soluble PD-L1 might be also a prognostic factor for various malignant tumors as high sPD-L1 level is associated with a poor prognosis [83,84,85].

Ding et al. [86] performed meta-analysis screening to evaluate the importance of sPD-L1 as a prognostic biomarker. Results from independent studies included samples of total 1102 patients with eight types of cancer, including NSCLC, multiple myeloma (MM), gastric adenocarcinoma (GA), and hepatocellular carcinoma (HCC), revealing a strong association between elevated levels of sPD-L1 and poor OS in all analyzed types of cancer.

Soluble PD-L1 has the ability to bind to mPD-1 [77]. It was hypothesized that blocking of mPD-1 by sPD-L1 results in resistance to anti-PD-1 therapy and that a high level of sPD-L1 in patients peripheral blood preceding anti-PD-1 therapy would be a potential negative predictive biomarker [87]. This hypothesis has a confirmation in the retrospective study of Okuma et al. [88] that included 39 NSCLC patients. High sPD-L1 plasma level before administration of nivolumab therapy was associated with shorter OS (7.20 months vs. not reached, *p* = 0.040) compared to patients with low sPD-L1 levels. Moreover, patients with low sPD-L1 levels had a better response (higher CR/PR/SD result) to therapy as compared to patients with high plasma sPD-L1 levels (higher PD response; *p* = 0.0069). High pre-treatment sPD-L1 concentration is also a negative prognostic biomarker in melanoma ICI therapy. Melanoma patients receiving ipilimumab or pembrolizumab monotherapies with a high sPD-L1 level had a worse prognosis in both treatment groups [81]. However, the correlation between early changes in concentration of sPD-L1 and treatment benefit in the anti-CTLA-4 or anti-PD-1 group was not confirmed. After 5 months of treatment, the elevation of sPD-L1 concentration correlated with partial responses to both ipilimumab- and nivolumab-treated patients [81]. In conclusion, it seems as though sPD-L1 has a predictive value, but its value is in the manner of its kinetic changes during treatment.

One of the many described tumor mechanisms of avoiding immune control is the release of exosomes [89]. Hypoxic conditions, temperature, and drug stress related to tumor environment were reported to intensify this process even more [90,91]. Extracellular vesicles (EVs) such as exosomes released by metastatic melanoma cells overexpressing PD-L1 were reported to carry PD-L1 bound to their surface that preserve its biological activity and bind to PD-1 on CTLS. Moreover, IFN-γ induces the secretion of exosomal PD-L1 (exoPD-L1) by melanoma cells [92]. These findings have opened the discussion about the prognostic and predictive potential of exoPD-L1 in melanoma therapies with ICI.

The plasma exoPD-L1 level is elevated in metastatic melanoma patients as compared to healthy donors. High pre-treatment exoPD-L1 concentration was observed in melanoma non-responders to pembrolizumab therapy and was associated with poorer clinical outcomes. However, more than a twofold increase of circulating exoPD-L1 within 6 weeks of therapy was related to better PFS and OS in the group of responders [92]. Cordonnier et al. [93], in contrast, reported no relationship between the pre-treatment exoPD-L1 level and OS or PFS in melanoma patients receiving anti-PD-1, ipilimumab, or anti-BRAF associated with anti-MAPK kinase (anti-MEK) treatment. However, a significant increase of exoPD-L1 was observed in patients experiencing the progression (*p* = 0.0002), regardless of the treatment. Moreover, changes in exoPD-L1 concentration during therapy were associated with patients’ response status. One of the main challenges in considering exoPD-L1 as a biomarker is setting cut-off values and sample collection time points. Inverse changes in exoPD-L1 concentration may predict similar outcomes for patients when samples are collected at different time points [92,93]. This intriguing observation could be explained by the ongoing development of the immune response to ICI therapy and changes in the tumor environment due to the release of pro-inflammatory cytokines.

Soluble CTLA-4 (sCTLA4) protein is a product of alternative splicing, one of the CTLA-4 isoforms. As a CTLA-4 receptor form, sCTLA-4 plays an important role in inhibitory regulation of T-cell-mediated immune response [94,95] and presents a predictive value in ipilimumab melanoma therapy. In two independent reports, high pre-treatment sCTLA-4 concentration was associated with a lower death rate and a favorable clinical outcome in melanoma patients receiving ipilimumab treatment [96,97].

Soluble CD25 (sCD25), a unit of the IL-2 receptor, was reported to bind directly to IL-2, affecting Treg augmentation [98,99]. High pre-treatment sCD25 concentration in metastatic melanoma patients before and during ipilimumab therapy correlated with poor outcomes. Patients with high sCD25 levels during therapy had worse OS compared to patients with low sCD25 concentration levels [99].

### 3.2. Peripheral Blood Biomarkers

#### 3.2.1. Total Cell Count and Ratios

Total cell count and ratios are routine peripheral blood laboratory analyses that are performed prior to the treatment as well as regularly during therapy. Such analyses can have significant importance for prognosis and response to ICI therapy. The neutrophil-to-lymphocyte ratio (NLR) is related to systemic inflammation, as an elevated number of neutrophils was reported in the peripheral blood of cancer patients [100,101]. High pre-treatment NLRs were related to worse OS for melanoma patients treated with ipilimumab (NLR > 3–5), nivolumab (NLR > 2–5), and the combination of nivolumab and ipilimumab (NLR > 4.7) [102,103,104,105,106,107]. Moreover, an increase of NLR over 30% during two cycles of anti-PD-1 treatment was related to lower OS in melanoma patients (47 vs. 13.5 months, *p* < 0.001) [108]. The absolute neutrophil count (ANC) and absolute lymphocyte count (ALC) were reported as early biomarkers related to OS in melanoma patients during nivolumab therapy. Additionally, a significantly increased OS was associated with ANC < 4000/µL and ALC ≥ 1000/µL levels after 6 weeks of treatment [70]. Over 1.35-fold ALC increase after two cycles of ipilimumab was related to a longer OS compared to patients with a lower increase [64]. Finally, the elevation of pre-treatment absolute or relative eosinophil counts (AEC or REC) was associated with better OS in melanoma patients receiving ipilimumab, pembrolizumab, or combined therapy with nivolumab and ipilimumab [18,107,109].

#### 3.2.2. MDSCs

Myeloid-derived suppressor cells (MDSCs) are a heterogeneous group of immature myeloid cells that are classified into two distinctive subpopulations: monocytic (mo-MDSC) and granulocytic (g-MDSC) cells. Pathological conditions such as cancer-associated chronic inflammation promote MDSC development, and an increased number of circulating MDSCs has a negative prognostic value in melanoma patients. The immunosuppressive potential of MDSCs to reduce the hosts’ anti-tumor immune response is related to inhibition of T-cell proliferation, CTL activity, and stimulation of the development of Tregs [110,111].

Patients with stage III and IV melanoma have elevated frequencies of circulating mo-MDSCs as compared to healthy donors and patients at the early stages of melanoma [112]. Low pre-treatment frequencies of mo-MDSCs were observed in responders to ipilimumab therapy and were associated with better survival. Furthermore, the decrease of mo-MDSCs as compared to the baseline was related to an increased OS and higher probability to benefit from therapy in patients after the first infusion [109,113,114,115]. Interestingly, Coana et al. [113] reported no correlation between mo-MDSC frequency and clinical benefit before treatment, but only during 3 weeks after the first ipilimumab infusion. Taken together, frequencies of circulating mo-MDSCs have a prognostic and predictive potential in melanoma therapy with anti-CTLA-4. Elevated pre-treatment frequencies of mo-MDSCs is a marker of a lower response, higher rates of progression, and shorter survival in melanoma patients receiving nivolumab who formerly progressed after ipilimumab therapy [116].

#### 3.2.3. Tregs

Tregs, defined as CD4⁺CD25⁺FoxP3⁺ cells, are a direct target of anti-CTLA-4 immunotherapy due to their constitutive CTLA-4 expression. The frequency of circulating Tregs is elevated in advanced melanoma patients compared to healthy donors. Moreover, an increase in Treg frequencies observed in these patients correlates with more advanced stages of the disease [112]. According to the basic understanding of the mechanisms of therapy with CTLA-4 antibodies, patients with high pre-treatment Treg frequencies would be a group who benefit the most from therapy. However, low pre-treatment Treg frequency was found to be associated with a favorable outcome and better OS following ipilimumab treatment [109] and was also correlated with no relapse in patients subjected to nivolumab adjuvant therapy [117]. Moreover, there are contradictory data about the correlation between the benefit of treatment and Treg changes during anti-CTLA-4 therapy. Simeone et al. [68] reported a significant decrease of Treg frequencies in patients with disease control and better OS at multiple time points during the first 12 weeks of therapy. In contrast, Tarhini et al. [118] observed an increase of Tregs and improved PFS in 6 weeks of neoadjuvant ipilimumab therapy. In agreement with this study, Woods et al. [119] reported increased Treg frequency in the group of melanoma patients responding to nivolumab therapy. The opposite changes in Treg frequency in the tumor microenvironment (TME) and in the blood that question their functional activity and increased phosphorylation of signal transducer and activator of transcription (STAT) in Tregs of responding patients can be a possible explanation of these contradictory findings [118,119]. Altogether these results suggest that not only the frequency of cells, but also their immunosuppressive potential and activity are relevant to the occurrence of response.

## 4. Predictive Biomarkers Related to the Host Gut Microbiota

The gut microbiota is a complex population of microorganisms that colonizes the mucosal surface of intestines [120]. Hugon et al. [121] identified over 2000 prokaryotic species in humans, however, intestinal mucosa is also inhabited by viruses and eukaryotic organisms, such as fungi or protozoans [122].

This numerous and diverse microbial population owns a large genomic content known as the microbiome and affects host organisms. Commensal gut microbiota play various roles, such as complex carbohydrate fermentation, short chain fatty acid (SCFA) production, mucosal barrier integrity strengthening, vitamin B and K synthesis, and protection against pathogens [120]. Moreover, another fundamental function of gut microbiota is the regulation of the mucosal and systemic immune system [123]. Gut-associated lymphoid tissue (GALT) is an integral part of the intestinal barrier and there is constant communication between gut microbiota and immune cells, and their interaction occurs through multiple pathways [124].

**Figure 2:**
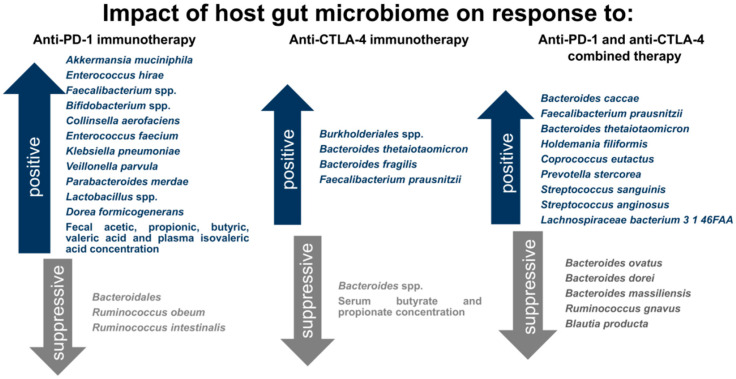
Beneficial and suppressive impact of gut microbiome on response to ICI therapy. Gut microbiota enriched with selected bacterial species is associated with enhanced antitumor responses and beneficial clinical outcome to ICI therapy, whereas other bacterial species suppress immune responses against cancer cells and are correlated with poor clinical outcome to ICI therapy.

Exploring the knowledge of gut microbiota significance led to the conclusion that it could affect drug metabolism. Studies devoted to various drug pharmacokinetics showed that drug activity and toxicity were correlated with gut microbiota composition. Some pathways of gut microbiota-mediated drug metabolism were identified, including the production of enzymes and other metabolites that directly affect drug molecules, as well as indirect modification of host genes involved in drug metabolism [125]. Therefore, the gut microbiota is also considered as a potential predictive biomarker of cancer treatment efficacy. For the first time, the association between gut microbiota and anticancer immunotherapy effects was shown by Viaud et al. [126] in 2013. In studies on cyclophosphamide on mouse models, the authors demonstrated that drug-stimulated alterations in intestinal microbiota composition and translocation of selected bacterial species, such as *Lactobacillus johnsonii*, *Lactobacillus murinus*, and *Enterococcus hirae* into secondary lymphoid organ stimulated antitumor immune responses, i.e., enhanced generation of T-helper 17 (T_H_17) cells and memory T_H_1 cells. However, germ-free (GF) mice and mice treated with antibiotics had a reduced the number of T_H_17 cells and were resistant to selected therapy. Additionally, growing evidence shows that gut microbiota also affects ICI therapy efficacy (Figure 2).

### 4.1. The Effects of Gut Microbiota on Anti-CTLA-4 Therapy Efficacy

Vétizou et al. [127] found that gut microbiota is essential in shaping the immune response to anti-CTLA-4 therapy against cancer cells. They observed that specific pathogen-free (SPF) mice responded to anti-CTLA-4 antibodies, whereas antibiotic-treated and GF mice were resistant to selected treatment. However, CTLA-4 blockade was restored in non-responding mice after recolonization of intestinal mucosa with selected *Bacteroides* and *Burkholderiales*. Moreover, analysis of gut microbiota composition of 25 melanoma patients treated with ipilimumab and fecal microbiota transplantation from patients to mice revealed that immunogenic *Bacteroides thetaiotaomicron* and *Bacteroides fragilis* elicited antitumor responses in mice. Studies showed that *B. fragilis* induced T_H_1 immune responses and maturation of DCs within tumors.

Chaput et al. [128], in studies on 26 patients with metastatic melanoma, demonstrated that patients’ baseline gut microbiota enriched with *Faecalibacterium* spp. and other genera belonging to the *Firmicutes* phylum was correlated with beneficial clinical outcome to ipilimumab. These patients had longer PFS and OS compared to those with a higher proportion of *Bacteroidetes* and more frequently had colitis as irAEs. Moreover, ipilimumab administration caused enhanced induction of inducible T-cell COStimulator (ICOS) on CD4^+^ T-cells and remarkable increase in serum CD25 in patients with *Faecalibacterium*-enriched microbiota. These results do not support previous findings [127]. However, limitations in the reconstitution of transplanted fecal microbiota in mice should be considered as one of the factors of such discrepancies [128]. Moreover, similar results were obtained Coutzac et al. [129] in studies on 38 fecal samples collected from metastatic melanoma. Gut microbiota enriched with *Faecalibacterium* spp. was linked with positive clinical responses, long-term PFS, and over 18 months of OS. Quantitative PCR revealed a positive correlation between the number of *Faecalibacterium prausnitzii* and bacterial load in fecal samples from patients with clinical benefits. Additionally, there was a higher number of *F. prausnitzii* in contrast to *B. fragilis* in patients with durable OS.

Bacterial metabolites, such as SCFAs, affect ICI therapy efficacy. However, there is a limited number of communications presenting this correlation. Coutzac et al. [129] demonstrated that anti-CTLA-4 therapy efficacy is inhibited by systemic SCFAs. They measured serum concentrations of three SCFAs, i.e., acetate, propionate, and butyrate in metastatic melanoma patients treated with ipilimumab (*n* = 85) and observed a negative correlation between serum levels of butyrate and propionate and clinical outcomes. Additionally, enhanced systemic SCFA concentration was associated with an elevated proportion of Tregs. Moreover, they analyzed the influence of serum butyrate concentration on anti-CTLA-4 therapy efficacy in mice supplemented with sodium butyrate. The treatment effects were also reduced in mice. They showed that systemic butyrate limited anti-CTLA-4 efficacy through inhibition of DC maturation, down-regulation of ICOS on T-cells, decrease of memory T-cell and antigen-specific T-cell expansion, negative modulation of CD28 signaling pathway, and IL-2 impregnation.

### 4.2. The Effects of Gut Microbiota on Anti-PD-1 Therapy Efficacy

Studies showed that selected species of gut microbiota also affect anti-PD-1 therapy efficacy in melanoma-bearing mice and melanoma patients. Various bacterial species were indicated as potential biomarkers of anti-PD-1 therapy efficacy in melanoma.

Routy et al. [130] showed that *Akkermansia muciniphila* and *Enterococcus hirae* were associated with antitumor response to anti-PD-1 therapy in mice and patients. For instance, this correlation was observed in RET mice (*ret* kinase transgenic mice spontaneously developing skin malignant melanoma) in SPF conditions (previously treated with broad-spectrum combination of antibiotics). Colonization of intestines with *A. muciniphila* separately or alongside *E. hirae* restored anticancer response to anti-PD-1 therapy in previously non-responding mice.

Gopalakrishnan et al. [131] conducted studies on 112 melanoma patients treated with anti-PD-1 therapy to examine differences in gut microbiota between responders and non-responders. Analysis of fecal samples revealed that gut microbiota composition of responding patients was more diversified and was enriched with bacteria belonging to the *Ruminococcaceae* family and the *Clostridiales* order, such as *Faecalibacterium* species, whereas non-responders represented low diversity and dominance of *Bacteroidales* in composition of their gut microbiota. Moreover, they demonstrated correlation between high abundance of the *Faecalibacterium* genus in gut microbiota and elevated levels of effector CD4^+^ and CD8^+^ T-cells in systemic circulation as well as infiltrating in the tumor along with maintained cytokine response. In contrast, *Bacteroidales* were associated with high levels of Tregs and MDSCs in systemic circulation and limited cytokine response. These findings suggest that gut microbiota regulates immune response to therapy against cancer cells. Fecal microbiota transferred from responders to GF mice also represented a high abundance of the *Faecalibacterium* genus and anti-tumor responses were also enhanced in these mice as compared to those with gut microbiota from non-responders.

Matson et al. [132] conducted studies on 42 metastatic melanoma patients treated with anti-PD-1 antibodies (*n* = 38) and anti-CTLA-4 antibodies (*n* = 4; results obtained for this cohort did not moderate general findings), among who 16 patients were responders and 26 patients were non-responders. They collected stool samples before treatment and performed identification of intestinal bacteria using DNA sequence-based techniques. Comprehensive analysis revealed that gut microbiota of responders was enriched with *Bifidobacterium longum*, *Bifidobacterium adolescentis*, *Collinsella aerofaciens*, *Enterococcus faecium*, *Klebsiella pneumoniae*, *Veillonella parvula*, *Parabacteroides merdae*, and *Lactobacillus* spp. They also found that *Ruminococcus obeum* and *Roseburia intestinalis* were more abundant in non-responders. Additionally, they transferred fecal microbiota from responders and non-responders to relevant cohorts of GF mice, and subsequently inoculated them with B16.SIY melanoma cells. In mice with successfully reconstituted microbiota from the responding donors, researchers observed slower baseline growth of tumor and enhanced frequency of SIY–specific CD8^+^ T-cells in the tumor microenvironment. Moreover, there was higher efficacy of anti-PD-L1 therapy in this mouse cohort, whereas it was ineffective in mice with fecal microbiota from non-responders.

Studies revealed that a high concentration of fecal SCFAs is positively correlated with clinical outcomes to treatment with anti-PD-1 therapy. Nomura et al. [133] conducted studies on 52 patients with solid tumors treated with nivolumab (*n* = 46) and pembrolizumab (*n* = 6), among whom melanoma patients constituted the largest cohort (*n* = 24). They observed response to anti-PD-1 therapy and notably longer PFS in patients with high concentrations of fecal SCFAs, i.e., acetic, propionic, butyric, and valeric acid and plasma isovaleric acid. Moreover, they noticed that frequent dietary fiber intake was associated with increased SCFA concentration in stool. Although the SCFA mode of action was not studied, researchers suggested that SCFAs modulate immune responses to anti-PD-1 therapy trough induction of FOXP3^+^CD4^+^ Treg differentiation; stimulation of specific molecules expression, such as IFN-γ, granzyme B, and IL-17; or histone deacetylase inhibition.

### 4.3. The Effects of Gut Microbiota on Combined Therapy with Anti-PD-1 and Anti-CTLA-4 Abs

Moreover, there have also been studies performed on patients treated with combined ICI therapies. Frankel et al. [134] demonstrated beneficial bacterial species that were associated with clinical response to ICI therapies in 39 patients with unresectable or metastatic melanoma. They analyzed microbial composition of stool samples collected from responders and non-responders using metagenomic shotgun sequencing. Studies showed that gut microbiota of all responding to ICI therapy patients was enriched with *Bacteroides caccae*. Additionally, *Faecalibacterium prausnitzii*, *Bacteroides thetaiotaomicron*, and *Holdemania filiformis* were found in patients with clinical response to combination therapy with nivolumab and ipilimumab, whereas *Dorea formicogenerans* was found in responders to pembrolizumab.

Peter et al. [135], in studies on 27 metastatic melanoma patients, also demonstrated the correlation between gut microbiota composition and ICI therapy effects (anti-PD-1 therapy, *n* = 14; anti-CTLA-4 therapy, *n* = 1; anti-PD-1/anti-CTLA-4 therapy, *n* = 12). *Coprococcus eutactus*, *Prevotella stercorea*, *Streptococcus sanguinis*, *Streptococcus anginosus*, and *Lachnospiraceae* bacterium 3 1 46FAA and previously found *Faecalibacterium prausnitzii* were identified in patients with longer PFS, whereas *Bacteroides ovatus*, *Bacteroides dorei*, *Bacteroides massiliensis*, *Ruminococcus gnavus*, and *Blautia producta* were present in the gut microbiota of patients with poor clinical outcomes.

According to the studies mentioned above, multiple bacterial species were indicated as potential predictive biomarkers of ICI therapy efficiency (Table 1). There were some discrepancies in results obtained for different cohorts of melanoma patients. However, numerous factors should be considered during comparison of received results, such as studied individuals, i.e., such as humans vs. animal models; studied material, i.e., differences in microbial composition of intestinal mucosa; and fecal content or other immune factors related to tumor or host. Nonetheless, a growing amount of evidence supports the view that gut microbiota and their metabolites play an essential role in shaping antitumor responses to ICI therapy, and this field should be explored to enhance clinical outcome. Recent observations of acquired resistance to ICI therapy may to some extent be hypothesized as a result of antibiotic therapy that has been introduced to treat infections or a change in diet factors that to a high extent influences composition, diversity, and function of microbiota.

**Table 1:** Summary of reviewed biomarkers and correlation with clinical benefit or response to ICI therapies for melanoma patients.

Biomarker	Treatment	Results (Correlation with Clinical Benefit or Response)	References
Tumor PD-L1 expression	Nivolumab	PD-L1⁺—better objective response	[21]
	Nivolumab/pembrolizumab	PD-L1⁺—better objective response,	[136]
	Pembrolizumab	PD-L1⁺—better response rate, PFS and OS	[27]
	Nivolumab + ipilimumab	Low PD-L1⁺—better survival outcome	[28]
Tumor MHC I/MHC II expression	Anti-PD-1/anti-PD-L1	High MHC II expression—improved clinical response, longer PFS and OS	[38]
	Ipilimumab/nivolumab	Reduced MHC I expression—worse OS, lack of clinical response >1% MHC II expression—complete or partial response, stabilization	[40]
Mutational load, neoantigen load	Ipilimumab/tremelimumab	>100 somatic mutations—clinical benefit of responders MHC I neoantigen response and survival	[46]
	Ipilimumab	>100 mutation load and high neoantigen load—better clinical benefit	[47]
	Anit-PD-1	Elevated mutational load—longer median PFS and OS	[38]
	Pembrolizumab/nivolumab	High mutation load—prolonged OS	[49]
ctDNA	Nivolumab/pembrolizumab	Pre-treatment undetected ctDNA—improved OS Decrease of ctDNA on-treatment—better PFS and OS	
	Ipilimumab, nivolumab, pembrolizumab, ipilimumab + pembrolizumab	Low pre-treatment ctDNA, on-treatment decrease of ctDNA—better response, prolonged PFS	[52]
	Anti-PD-1	Pre-treatment undetected ctDNA—better PFS and OS Pre-treatment or early on-treatment > 500 ctDNA copies—poor clinical outcome	[53]
Total cell count and ratios	Ipilimumab, nivolumab Ipilimumab + nivolumab	High NLR ratio—worse OS	[102,103,104,105,106,107]
	Nivolumab	>30% increase NLR ratio on-treatment—lower OS	[108]
	Nivolumab	<4000/µL ANC, ≥1000/µL ALC on-treatment—increase OS	[70]
	Ipilimumab	>1.35-fold increase ALC on-treatment—longer OS	[64]
	Ipilimumab, pembrolizumab, nivolumab + ipilimumab	Elevated AEC on-treatment—better OS	[18,107,109]
Mo-MDSCs	Ipilimumab	Elevated mo-MDSCs—non responders, worse survival Decrease mo-MDSsC on-treatment—increased OS, higher probability of benefit from therapy	[109,113,114,115]
Tregs	Ipilimumab	Low Tregs—favorable outcome, better OS	[113]
	Nivolumab adjuvant therapy	Low Tregs—no relapse	[117]
	Ipilimumab	Decrease Treg son-therapy—disease control, better OS	[68]
	Ipilimumab neoadjuvant	Increase Tregs—improved PFS	[118]
	Nivolumab	Increase Tregs—responding	[119]
Cytokines	Nivolumab	High pre-treatment IFN-γ, IL-6, IL-10—response Decrease IFN-γ, IL-6, IL-10—no response	[74]
	Nivolumab	Elevated pre-treatment IL-6—irAE occurrence	[75]
	Anti-CTLA-4 + anti-PD-1	Elevated G-CSF, GM-CSF, Fractalkine, FGF-2, IFN-α2, IL-12p70, IL-1a, IL-1B, IL-1RA, IL-2 and IL-13—irAE occurrence	[76]
Soluble checkpoint molecules	Nivolumab	High sPD-L1—shorter OS, worse response	[88]
	Ipilimumab/pembrolizumab	High sPD-L1—worse prognosis Elevation sPD-L1 on-treatment—partial response	[81]
	Pembrolizumab	High pre-treatment exoPD-L1—no response, poor clinical outcome >2-fold increase on-treatment—better PFS and OS in responders	[92]
	Ipilimumab/anti-BRAF + anti-MEK	Elevation exoPD-L1 on-treatment—progression	[93]
	Ipilimumab	High sCTLA-4—favorable clinical outcome, low death rate	[96,97]
	Ipilimumab	High sCD25—poor outcome, worse OS	[99]
LDH	Ipilimumab Nivolumab/pembrolizumab Nivolumab + ipilimumab	Elevated LDH—worse outcome, shorter OS	[61,62,63,64,65]
CRP	Ipilimumab	decrease CRP—response, stabilization and survival	[68,69]
	Anti-PD-1	elevated LDH—poor OS	[70]
S100B	Pembrolizumab Nivolumab + ipilimumab	Elevated S100B—impaired OS	[63]
Selected bacterial species	Anti-CTLA-4	*B. thetaiotaomicron*, *B. fragilis*, *Burkholderiales*—antitumor response	[127]
	Ipilimumab	*F. prausnitzii* and bacterial load—beneficial clinical outcome, longer PFS and OS	[128,129]
	Ipilimumab	*Bacteroides* spp.—poor clinical outcome	[128,129]
	Anti-PD-1	*A. muciniphila*, *E. hirae*, *Faecalibacterium* spp., *Bifidobacterium* spp., *C. aerofaciens*, *E. faecium*, *K. pneumoniae*, *V. parvula*, *P. merdae*, *Lactobacillus* spp., *D. formicogenerans*—antitumor responses	[130,131,132,134]
	Anti-PD-1	*Bacteroidales*, *R. obeum*, *R. intestinalis*—poor clinical outcome	[131,132]
	Combined ICI therapies	*B. caccae*, *F. prausnitzii*, *B. thetaiotaomicron*, *H. filiformis*, *C. eutactus*, *P. stercorea*, *S. sanguinis*, *S. anginosus*, *L. bacterium 3 1 46FAA*—beneficial clinical outcome	[134,135]
	Combined ICI therapies	*B. ovatus*, *B. dorei*, *B. massiliensis*, *R. gnavus* and *B. producta*—poor clinical outcome	[135]
Microbial metabolites	Ipilimumab	Elevated serum levels of propionate and butyrate—poor clinical outcome	[129]
	Nivolumab/pembrolizumab	elevated fecal SCFAs, i.e., acetic, propionic, butyric, and valeric acid and plasma isovaleric acid—response to therapy and longer PFS	[133]

## 5. Conclusions

Recently, the availability of various treatment options has become a fact, mainly because of the approval of novel immunotherapies for melanoma. However, some important issues remain to be resolved, for instance, the choice of accurate, specific, and optimized biomarkers of response to ICI therapies. Biomarkers are useful tools that simplify the decision-making of first-line treatment and can elevate therapy to the personalized level. Moreover, the limitation of damages related to undesirable adverse events would be also beneficial. Several potential biomarkers related to the tumor, the host immune system, and the host gut microbiota have been proposed, but still none of them are officially recommended for melanoma immunotherapy. Apparently analyzing more than one type of biomarker is the answer to this issue, because of the extended context that takes into account the properties of the tumor and the patient’s response potential, and does not ignore its microflora composition. Before starting treatment, analyzing the properties of the tumor (such as PD-L1, MHC I, and II expression status) would help to make a decision about the choice of the type of immunotherapy. Moreover, collecting information about patients’ immune potential in terms of responding to the treatment such as diversity of the intestinal microflora and circulating biomarkers in whole blood (such as soluble checkpoint molecules, cytokines, and ctDNA levels) are necessary to monitor the patients’ responses to the applied treatment, and further for early detection of secondary resistance to therapy. Because of the growing understanding of the complexity of immunomodulatory mechanisms in cancer development, modern immunotherapy is moving towards combinational therapies. In turn, these treatment strategies demand the exploration of the knowledge of novel and relevant predictive biomarkers.
